# The Clinical Pharmacology of Intranasal l-Methamphetamine

**DOI:** 10.1186/1472-6904-8-4

**Published:** 2008-07-21

**Authors:** John E Mendelson, Dana McGlothlin, Debra S Harris, Elyse Foster, Tom Everhart, Peyton Jacob, Reese T Jones

**Affiliations:** 1 Addiction Pharmacology Research Laboratory, The California Pacific Medical Center Research Institute, St. Luke's Hospital, 7th floor, 3555 Cesar Chavez Street, San Francisco, CA 94110, USA; 2 Drs. McGlothin and Foster: Department of Cardiology, University of California, San Francisco, 401 Parnassus Avenue, San Francisco, CA 94143-0984, USA; 3 Department of Psychiatry, University of Cincinnati and Cincinnati VA Medical Center, 3200 Vine St Cincinnati, Ohio, 45220, USA; 4 Department of Psychiatry, University of California, San Francisco, 401 Parnassus Avenue, San Francisco, CA 94143-0984, USA

## Abstract

**Background:**

We studied the pharmacology of l-methamphetamine, the less abused isomer, when used as a nasal decongestant.

**Methods:**

12 subjects self-administered l-methamphetamine from a nonprescription inhaler at the recommended dose (16 inhalations over 6 hours) then at 2 and 4 (32 and 64 inhalations) times this dose. In a separate session intravenous phenylephrine (200 μg) and l-methamphetamine (5 mg) were given to define alpha agonist pharmacology and bioavailability. Physiological, cardiovascular, pharmacokinetic, and subjective effects were measured.

**Results:**

Plasma l-methamphetamine levels were often below the level of quantification so bioavailability was estimated by comparing urinary excretion of the intravenous and inhaled doses, yielding delivered dose estimates of 74.0 ± 56.1, 124.7 ± 106.6, and 268.1 ± 220.5 μg for ascending exposures (mean 4.2 ± 3.3 μg/inhalation). Physiological changes were minimal and not dose-dependent. Small decreases in stroke volume and cardiac output suggesting mild cardiodepression were seen.

**Conclusion:**

Inhaled l-methamphetamine delivered from a non-prescription product produced minimal effects but may be a cardiodepressant.

## Background

There are two enantiomers of methamphetamine: dextrorotatory (d) methamphetamine and levorotatory (l) methamphetamine. The d-isomer is commonly abused and is available by prescription (DEA Schedule II), but unknown to most physicians the l-isomer is sold over-the-counter and is the active ingredient of the Vicks^® ^Vapor Inhaler (spelled levmetamfetamine by the manufacturer, Procter & Gamble, Cincinnati, OH) [1,2]. Each Vicks inhaler contains about 50 mg of l-methamphetamine, and earlier estimates suggested delivered daily doses between 1.9 to 7.2 mg of drug when used as directed [3]. The over-the-counter (OTC) vasoconstrictor nasal decongestant phenylpropanolamine (PPA) was associated with an increased risk of hemorrhagic stroke in young women that led to it being voluntarily withdrawn from the market [4]. Ephedra, another component of OTC nasal decongestants may also increase the risk of cardiovascular adverse events [5]. Methamphetamine is a sympathomimetic vasoconstrictor that increases blood pressure and myocardial oxygen consumption [6].

Despite the widespread use of OTC nasal decongestants, there is surprisingly little published data on their pharmacologic effects. Thus, physicians may encounter patients using l-methamphetamine but have little data on risks and drug exposure. To date, there are no published data on the pharmacology of intranasal l-methamphetamine. The complications of OTC decongestants, although severe, are exceedingly rare. Because adverse events of these drugs are uncommon, observational studies are unlikely to delineate mechanisms of action or toxicity. Measures that assess cardiovascular, and pharmacokinetic variables could assist in predicting risk and defining mechanisms of action.

The effects of l-methamphetamine on cardiac function are not yet defined. In this study we used impedance cardiography to measure the effects of methamphetamine-induced vasoconstriction on vascular resistance and cardiac work. Because people using the inhaler are likely to exercise (indeed one Olympic athlete lost a medal due to Vicks inhaler use before his race) [7], interactions between exercise and intranasal methamphetamine were assessed. Understanding the effects of intranasal l-methamphetamine contained in the Vicks^® ^Inhaler is important due to the associated cardiovascular risks of other OTC nasal decongestants. In addition, establishing the relative and absolute bioavailability of l-methamphetamine in the Vicks^® ^Inhaler would aid in a further assessment of risk. In this study, we examined the pharmacokinetic, cardiovascular, and subjective effects of l-methamphetamine. Doses were delivered using an easily available non-prescription product – the Vicks^® ^Vapor Inhaler.

## Methods

### Subject selection

Twelve subjects with a mean age of 38 (range 28 to 51 years old) participated in the study. Subjects were recruited through advertisements, and were included if they were normotensive, between 18 to 65 years, had a normal physical examination, EKG, blood and urine chemistries and had no nasal pathology that might alter the absorption of l-methamphetamine.

Subjects were excluded if they had used nasal decongestants within the last three months, were dependent on any drugs other than caffeine or nicotine or had structural abnormalities of the heart seen during a preliminary stress echocardiogram. Women were required to have a negative serum pregnancy test (Unilab, San Jose, California) before each session. Subjects were not dependent on methamphetamine, alcohol or other illicit drugs using DSM-IV-R criteria.

Because the Vicks^® ^Inhaler is an OTC product, prior experience with methamphetamine was not necessary. Informed consent was obtained and subjects were paid for their participation. The study was approved by the UCSF IRB.

### Study design

Inhaled l-methamphetamine was self administered from the commercially available Vick's^® ^Inhaler using a 3 session, ascending dose, open-label study design with sessions separated by at least 1 week. Dosing was starting at the manufacturer's recommended dose of 2 inhalations per nostril every 2 hours. Within each session 4 dosing periods occurred with periods separated by 2 hours; all dosing within a session occurred over 8 hours. In the first session subjects received 4 inhalations per period. Thus, over 8 hours a total of 16 inhalations were given. In the second and third sessions, the inhaler was administered with the same 2 hour dose period but at 2 and 4 times the recommended dose. In these sessions a total of 32 and 64 inhalations were given. Inactive ingredients in the Vicks inhaler include bornyl acetate, camphor, lavender oil, menthol and methyl salicylate. Subjects were admitted as inpatients to the UCSF General Clinical Research Center (GCRC) for approximately 36 hours.

Following administration of 3 ascending dose inhalations, a fourth session was conducted to contrast the effects of a prototypic alpha agonist phenylephrine, (Gensia Sicor Pharmaceuticals, Irvine, CA) with those produced by inhaled methamphetamine and to administer a parenteral dose of l-methamphetamine that would allow determination of absolute bioavailability. A similar phenylephrine challenge procedure has been safely used to measure hemodynamic response in hypertensive patients maintained off all antihypertensive medication for 2 weeks [8]. The phenylephrine response test was performed by giving an intravenous phenylephrine bolus dose of 100 μg. If the initial dose did not produce a 15 mm rise in systolic blood pressure within 15 minutes, a second 200 μg dose was given at 30 minutes. Two hours after the last phenylephrine dose, a slow (over 15 minutes) intravenous infusion of 5 mg of l-methamphetamine was administered to establish absolute bioavailability. The l-methamphetamine was prepared from the free base obtained from Sigma (St. Louis, MO) under FDA IND 58,189. We had planned to quantify relative bioavailability by extracting and quantifying the residual l-methamphetamine content from the inhalers. However, the amount of l-methamphetamine in new inhalers was highly variable (between 50 and 75 mg) and, given the small delivered doses, we were not able to obtain sufficiently accurate inhaler weights before and after dosing to allow estimation of delivered doses.

Subjects were requested to abstain from both nicotine and caffeine for approximately 12 hours before each dosing and from alcohol for 48 hours prior to dosing. Subjects were excluded from participation if they had a positive qualitative urine test for abused drugs prior to dosing or reported use of nicotine, caffeine or alcohol with the above windows; no subjects were excluded for positive qualitative urinalysis or caffeine, nicotine or alcohol use within specified time limits. No caffeine was allowed until stress echocardiograms were completed. No smoking was permitted during the hospital stay; nicotine patches were offered to all smokers. Vicks^® ^Inhalers from a single batch were used for each subject; all inhalers were purchased from a single pharmacy.

## Measures

### Physiological measures

Blood pressure, heart rate, skin and core temperature, and respiratory rate were measured using a non-invasive automatic device (Escort II 300 Patient Monitor, Medical Data Electronics, Arleta, Calif.) at 15 minutes before and 5, 15, 30 and 60 minutes within each period; and 2, 4, 8, 18, 24, and 30 hours after the last period to assess safety. For Session 4, before and after intravenous phenylephrine and l-methamphetamine, vital signs were measured at 5-minute intervals until they returned to baseline, then hourly for 8 hours, and then at 8-hour intervals until discharge.

### Echocardiography and stress-echocardiograph

2D echo-Doppler examination was performed at the time of enrollment to ensure normal cardiac structure and function. To determine if l-methamphetamine produces alterations in myocardial contractility with changes in vascular resistance, a treadmill stress echocardiogram was performed 15 minutes following the last period, except after the methamphetamine infusion (session 4) where the echo was done at 30 minutes. Subjects exercised to general fatigue or until asked to stop. Stress ECG results were defined as abnormal if there was ≥ 1.0-mm ST-segment depression measured at 80 ms after the J point in 2 contiguous leads during peak stress or immediately after recovery.

Stress echocardiographic measurements were made at baseline and 50%, 70%, and 85% of maximal heart rate. The cardiovascular variables measured by stress echocardiography included systolic blood pressure, heart rate, cardiac output, stroke volume, ejection fraction, systolic wall stress, septal wall thickness, posterior wall thickness, and left-ventricular internal diameter. Measurements obtained approximately 15 minutes before exercise, and immediately following exercise (85% of maximal heart rate) were used in final analyses. Echocardiographic data was analyzed offline (ProSolv CardioVascular Analyzer with DICOM software).

### Impedance cardiography

Impedance cardiographic measures of stroke volume and ejection times were obtained before and at 15 minutes and 2 hours within each period. Blood pressure and heart rate were simultaneously obtained. From these variables, systemic vascular resistance, left cardiac work, left ventricular ejection time, and cardiac output were determined using a dedicated commercially available system (Cardiodynamics) consisting of a personal computer with customized data processing software, a transmitting unit with four pairs of electrodes for analyses of the thoracic impedance field.

### Biological samples

#### Blood samples

For Sessions 1 to 3, 5-ml plasma samples for methamphetamine and metabolites were obtained 15 minutes after inhalations (theoretical peak) and 5 minutes before the next series of inhalations (theoretical trough), and then 4, 8, 18, 24, and 30 hours after the last inhaled dose. For Session 4, samples were obtained before and at 30 minutes, 1, 2, 4, 8, 18, 24, and 30 hours after methamphetamine. Samples were placed on ice immediately after collection. Samples were obtained through an in-dwelling venous catheter using sterile technique.

#### Urine samples

All urine was collected for the time from admission to just prior to dosing and from 0–12 hours, 12–24 hours, and 24–36 hours after the beginning of dosing.

#### Assays

Plasma and urine l-methamphetamine concentrations were determined according to a previously described gas chromatography-mass spectrometry method [9].

### Subjective measures

Visual Analog Scales were administered (Sessions 1 to 3) 15 minutes before the first period, at 5 and 30 minutes within each period, and then 4, 8, 18, and 30 hours after the last l-methamphetamine dose. In Session 4, tests were administered 15 minutes before the phenylephrine dose and then 0.5, 1.5, 4, 8, 18, and 30 hours after the first phenylephrine dose. Items included Visual Analog Scale ratings of "any drug effect," "good drug effect," "bad drug effect," "nasal stuffiness," "nasal dryness," "headache," and "dizziness." Visual analog ratings were performed by asking the subject to place a vertical mark along a 100 mm line with 0 defined as "none" and 100 as the "most ever."

### Statistical analysis

Group comparisons with PC-SAS's general linear model procedure (SAS Institute Inc., Release 6.04 Edition, Cary, N.C., 1990) and with multifactor repeated-measures analysis of variance were done with SAS (UNIX) or Super ANOVA (Macintosh) software applications. Physiologic data were transformed to change scores (post-treatment minus pre) and analyzed by repeated measures analysis of variance (ANOVA). Each session had 4 dosing periods. Within each period the mean value of the identical time points were calculated and used in the analysis. After a significant F-test, pair-wise comparisons were performed using the least squares means analysis. Effects were considered statistically significant at p ≤ 0.05. Data is presented as mean (SD).

## Results

### Methamphetamine concentrations

**Figure 1:**
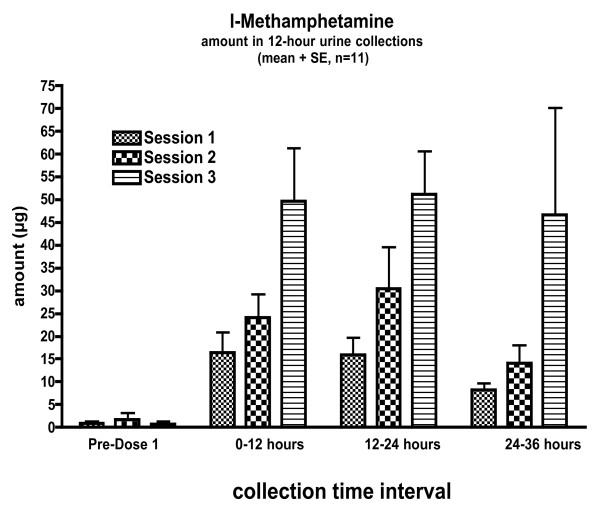
Methamphetamine and amphetamine concentrations excreted in the urine from 0–12, 12–24, and 24–36 hours. Values are means (SE). N = 11.

Plasma methamphetamine and amphetamine concentrations were often below the limit of quantification (< 5 ng/ml). Thus, absolute bioavailability and pharmacokinetic variables could not be calculated. Measurable quantities were excreted in the urine with maximum levels at the highest dose (4× the recommended dose), showing a dose response to inhalations. Peak amounts excreted in urine occurred between 12–24 hours and then decreased significantly during the 24–36 hour collection (Figure 1).

We estimated the dose delivered by comparing urinary excretion from the three inhalation conditions with the iv condition. Total methamphetamine excretion (0–36 hours) was 40.7 (30.9) μg, 68.6 (58.6) μg, and 147.4 (121.2) μg for the 16, 32 and 64 inhalation conditions. It was 2749.5 (499.6) μg following the 5 mg IV dose. Assuming similar distribution and elimination of inhaled and intravenous doses, estimated delivered nasal doses for each session are 74.0 (56.1) μg, 124.7 (106.6) μg, and 268.1 (220.5) μg, respectively. The estimated delivery of a single inhalation is approximately 4.2 (3.3) μg per inhalation (range 0.8–14.3 μg/inhalation). Following inhalations approximately 4% of the dose was excreted as l-amphetamine (corrected for difference in molecular weight); after intravenous dosing approximately 3% of the dose was excreted as l-amphetamine.

### Physiological measures

Most physiological variables did not change in a clear dose-dependent manner. For example, systolic blood pressure increased by 11.8 (16.2) and 12.3 (20.5) mmHg (p = 0.02) in the 16 and 32 but fell by 1.2 (16) mmHg 64 inhalation conditions. Mean peak diastolic blood pressure increased by 7 to 9 mmHg (p = 0.04) with no difference between doses. Across time, core temperature increased by ~0.1°C in the 16 and 32 inhalation conditions and decreased by ~0.1°C in the 64 inhalation condition (p = 0.02). In the 64-inhalation condition respiratory rate increased by 0.4 (1.68) (p = 0.02) breaths per minute; no hyperthermia or respiratory distress was seen in any condition. Peak respiratory rate increased by a clinically insignificant 3 (2.5) breaths per minute in the 32-inhalation condition (p = 0.03). No significant increases in heart rate were seen.

The intravenous methamphetamine dose did not alter cardiovascular parameters – mean (SD) peak responses were 2.9 (9.3) mmHg, 7.4 (12.5) mmHg, and 0.42 (4.4) breaths/min in systolic and diastolic blood pressure, and respiratory rate, respectively. In contrast to the results seen in hypertensives, the phenylephrine doses (100 and 200 μg) produced no significant changes in blood pressure or heart rate (Table 1). Interestingly, the 16 and 32 inhalation conditions produced substantially more robust effects on systolic blood pressure than the much larger intravenous l-methamphetamine and phenylephrine doses. All three inhalation conditions increased diastolic blood pressure more than phenylephrine or intravenous l-methamphetamine. Mean peak changes in physiological variables are shown in Table 1.

**Table 1:** Peak Changes in Physiological Variables in Response to Inhaled l-Methamphetamine, IV phenylephrine and IV l-methamphetamine.

Measure	16 Inhalations	32 Inhalations	64 Inhalations	PE_1_	PE_2_	IV l-Meth	Overall P-Value
Systolic Blood Pressure (mmHg)	11.8 (16.2)^‡§||^**	12.3 (20.5)^‡§||^**	- 1.2 (16.0)	0.9 (10.1)	1.4 (9.8)	2.9 (9.3)	0.03
Diastolic Blood Pressure (mmHg)	8.7 (8.9)^||^**	6.6 (9.9)^||^	9.0 (12.4)^||^**	1.9 (8.3)	-2 (10.2)	7.4 (12.5)^||^	0.04
Heart Rate (beats/min)	1.3 (12.3)	4.2 (19.2)	2.3 (14.3)	2.8 (9.7)	-4.2 (7.9)	- 0.33 (8.4)	0.62
Respiratory Rate (breaths/min)	0.17 (4.3)	3 (2.5)*^§||^**	2.4 (3.5)^§||^	- 1.9 (4.8)	-1.5 (3.3)	0.42 (4.4)	0.003
Core Temperature (°C)	0.13 (0.8)	- 0.32 (0.9)	0.21 (0.9)	- 0.19(0.6)	0.08 (0.4)	0.09 (0.2)	0.41

Data presented as mean (SD).

PE_1 _= phenylephrine dose 1; PE_2 _= phenylephrine dose 2; IV l -Meth = IV l-methamphetamine

* = significantly greater than 16 inhalations

† = significantly greater than 32 inhalations

‡ = significantly greater than 64 inhalations

§ = significantly greater than PE_1_

|| = significantly greater than PE_2_

¶ significantly greater than IV l-methamphetamine

** = significantly greater than baseline

### Stress echocardiography

Intranasal l-methamphetamine did not alter the effect of exercise on most cardiovascular measures. Exercise produced expected increases in cardiac output (p < 0.001), ejection fraction (p < 0.001), heart rate (p < 0.001), systolic wall stress (p = 0.002), and systolic blood pressure (p ≤ 0.001) and expected decreases in end-systolic left ventricular internal diameter (p = 0.01). The cardiac response to exercise was not affected by any inhaler dose level except for septal wall thickeness (SWT), which increased significantly only after the highest inhaler dose. This difference is most likely due to a single outlier. Echocardiographic measurements are shown in Table 2.

**Table 2:** Change in Cardiovascular Variables in Response to Exercise After Vicks Inhalation and IV l-Methamphetamine As Measured by Stress Echocardiography. Data presented as mean (SD).

Measure	16 Inhalations	32 Inhalations	64 Inhalations	IV l-Meth	Overall P-Value (time)	Overall P-Value (dose)
Cardiac Output (l/min)	3.1 (2.0)	2.4 (2.2)	2.5 (2.5)	3.8 (2.3)	< 0.001	0.10
Heart Rate (beats/min)	73.5 (26.7	66.3 (21.5)	64.5 (22.9)	76.9 (16.8)	< 0.001	0.17
Systolic Blood Pressure (mmHg)	41.3 (18.7)	41.6 (42.6)	52.0 (13.9)	57.6 (11.6)	< 0.001	0.35
Stroke Volume (ml)	3.3 (13.2)	2.5 (13.4)	2.1 (13.9)	-0.02 (7.3)	0.41	0.90
LV Internal Diameter (cm)	-2.6 (2.9)	-2.1 (3.5)	-1.8 (4.1)	-2.0 (4.6)	0.01	0.96
Ejection Fraction (%)	8.9 (5.8)	7.8 (7.6)	6.3 (6.4)	9.9 (6.5)	< 0.001	0.62
Systolic Wall Stress(kdynes/cm^2^)	64.5 (62.8)	51.7 (61.6)	41.7 (79.3)	81.7 (90.6)	0.002	0.42
Posterior Wall Thickness (cm)	- 0.58 (1.4)	0.67 (1.3)	0.25 (0.97)	0.08 (1.5)	0.49	0.20
Septal Wall Thickness (mm)	0.08 (0.79)	0.08 (1.5)	2.0 (3.1)	0.25 (0.97)	0.05	0.04

Responses are post-exercise minus pre; wall thicknesses measurements are end-systolic

LV = Left Ventricular

p-values are based on overall p-values across time and dose.

### Impedance cardiography

Significant differences were seen in a few cardiovascular parameters at 15 minutes post dose. Inhaled l-methamphetamine decreased stroke volume by 3.9 to 6 mls/beat (p = 0.01). Heart rate fell slightly by ~1 to 2 beats/per minute. These small decreases in heart rate and stroke volume decreased cardiac output (CO) by ~0.5 l/min (p = 0.02). Systemic vascular resistance (SVR) increased in all conditions by 106 to 137 dynes*sec*cm^5 ^(1190 dynes*sec*cm^5^, 1259 dynes*sec*cm^5^, and 1278 dynes*sec*cm^5 ^for 16, 32 and 64 inhaler doses, p = 0.004). However, this was a small absolute increase of less than 10%. The increase in SVR is probably compensating for the decrease in CO, and was not accompanied by an increase in blood pressure.

At two hours post-dose, many parameters were significantly different from pre-dose conditions. In response to l-methamphetamine, stroke volume remained 1.6 to 6.2 mls/beat below baseline (p = 0.02). Heart rate increased by 4 to 5 beats/min (p = 0.003). Therefore, cardiac output returned to approximately pre-dose values. Diastolic blood pressure increased slightly by ~2 mmHg (p = 0.01). A decrease of ~12 ms occurred in left ventricular ejection time possibly resulting from the increase in heart rate. No significant differences were found between conditions; parameters are shown in Table 3. These results suggest that l-methamphetamine has mild cardiodepressant actions that initiate compensatory increases in heart rate and systemic vascular resistance.

**Table 3:** Change in Cardiovascular Variables in Response to Inhaler Condition and IV l-Methamphetamine as Measured by Impedance Cardiography.

Measure	16 Inhalations	32 Inhalations	64 Inhalations	IV l-Meth	Overall P-Value (time)	Overall P-Value (dose)
Cardiac Output (l/min)						
15 minutes	-0.53 (0.66)	-0.46 (1.3)	-0.56 (0.52)	-0.01 (0.54)	0.02	0.67
2 Hours	0.0 (0.67)	0.05 (1.6)	0.31 (0.77)	0.28 (1.0)	0.32	
Heart Rate (beats/min)						
15 Minutes	-2.6 (4.9)	-2.1 (5.8)	-1.7 (5.9)	1.0 (5.8)	0.38	0.60
2 Hours	4.3 (6.0)	5.4 (11.3)	4.3 (7.7)	6.1 (9.5)	0.003	
Systolic Blood Pressure (mmHg)						
15 Minutes	1.0 (12.5)	-1.2 (6.6)	-3.0 (6.4)	4.8 (9.0)	0.76	0.09
2 Hours	1.8 (7.9)	-0.17 (7.0)	3.1 (6.9)	5.9 (6.9)	0.06	
Diastolic Blood Pressure (mmHg)						
15 Minutes	0.50 (7.5)	1.4 (6.7)	1.2 (6.4)	4.3 (8.8)	0.09	0.23
2 Hours	2.2 (4.2)	2.6 (6.5)	1.2 (4.9)	6.5 (8.1)	0.007	

Stroke Volume						
15 Minutes	-3.9 (7.3)	-4.9 (16.0)	-6.0 (6.4)	-1.0 (5.4)	0.008	0.66
2 Hours	-5.4 (6.6)	-6.2 (17.5)	-1.6 (7.8)	-0.67 (6.4)	0.02	
Systemic Vascular Resistance (dynes*sec*cm^5^)						
15 Minutes	106.0 (168.9)	137.1 (388.5)	129.1 (169.5)	48.8 (112.7)	0.004	0.89
2 Hours	47.2 (140.2)	72.0 (441.7)	-10.2 (204.0)	12.3 (127.2)	0.36	
Left CardiacWork						
15 Minutes	-0.51 (1.0)	-0.48 (1.6)	-0.59 (0.64)	0.44 (0.93)	0.21	0.12
2 Hours	0.22 (0.91)	0.23 (2.1)	0.53 (0.78)	1.1 (1.5)	0.03	
LV Ejection Time						
15 Minutes	-0.75 (12.4)	-4.8 (16.4)	-12.4 (19.1)	-0.25 (10.4)	0.19	0.54
2 Hours	-11.8 (16.4)	-12.1 (26.8)	-11.8 (19.9)	-6.4 (26.5)	0.005	

Data presented as mean (SD).

IV-l-meth-IV l-methamphetamine; p-value (time)-compared to baseline vs. 15 minutes and 2 hours post-dose

### Subjective ratings

The subjective effects of inhaled l-methamphetamine were modest. In all three inhaler conditions VAS peak (p < 0.01) and overall (p = 0.001) ratings of "Any Drug Effect increased, indicating that subjects noted a drug effect. However, other ratings were inconsistent. VAS "Bad Drug Effect," (p = 0.01) and "Dizziness" (p = 0.002) both significantly increased across time with no significant differences between inhaler conditions but peak effects were trivial and non-significant. The 64 inhalation condition increased peak "Good Drug Effect" (p = 0.001) but only to 9.7 (13.0) on a 0–100 VAS scale. The 64 inhalation condition increased VAS "headache" (p = 0.01) but again the effect was modest [12.7 (17.0)]. Interestingly, the parenteral phenylephrine and l-methamphetamine produced less subjective effects than the inhaled l-methamphetamine doses. Mean peak changes are shown in Table 4.

**Table 4:** Peak Changes in VAS Subjective Variables in Response to Inhaled l-Methamphetamine, IV phenylephrine and IV l-methamphetamine.

Measure	16 Inhalations	32 Inhalations	64 Inhalations	PE_1_	PE_2_	IV l-Meth	Overall P-Value
Any Drug Effect	9.0 (13.9)^§||^**	7.3 (10.5)^§||^**	9.8 (7.9)^§||¶^**	1.5 (2.1)	1.7 (2.4)	4.5 (8.6)	0.001
Bad Drug Effect	6.8 (13.7)	2.8 (4.6)	4.9 (5.0)	0.33 (0.9)	1.1 (0.79)	5.3 (10.7)	0.07
Dizziness	9.3 (14.0)^§||¶^**	6.5 (6.4)^¶^**	9.0 (13.5)^§||¶^**	1.5 (2.9)	0.92 (2.2)	-0.67 (5.7)	0.004
Good Drug Effect	5.8 (13.0)^||^**	5.7 (10.5)**	9.7 (13.0)^§||¶^**	0.42 (2.9)	0.25 (1.5)	2.7 (4.7)	0.005
Headache	5.1 (18.1)	4.8 (5.1)^||^	12.7 (17.0)^§||¶^**	-0.08 (1.7)	1.8 (3.3)	-3.8 (13.4)	0.02
Nasal Dryness	5.2 (16.2)	3.3 (9.6)	4.1 (9.9)	-0.75 (2.1)	-0.50 (1.7)	-0.42 (2.7)	0.20
Nasal Stuffiness	1.1 (16.9)	2.8 (4.8)	1.4 (6.7)	0.50 (4.4)	-1.6 (3.6)	0.42 (1.2)	0.88

Data presented as mean (SD).

PE_1 _= phenylephrine dose 1; PE_2 _= phenylephrine dose 2; IV l -Meth = IV l-methamphetamine

*= significantly greater than 16 inhalations

† = significantly greater than 32 inhalations

‡ = significantly greater than 64 inhalations

§ = significantly greater than PE1

|| = significantly greater than PE2

¶ significantly greater than IV l-methamphetamine

** = significantly greater than baseline

## Discussion

l-Methamphetamine delivered through the Vicks^® ^Inhaler was well tolerated and produced minimal pharmacodynamic effects, even at 4 times the maximum recommended dose. Dose dependent but small increases in systolic and diastolic blood pressure were seen. Impedance cardiography results suggest that l-methamphetamine may actually depress cardiac function. There were no effects of increasing l-methamphetamine dose on stress echocardiography. Small increases in visual analog "Good Drug Effect" were seen (from a mean of 5.8 to 9.7) but increases of a similar magnitude in ratings of "Bad Drug Effect," and "Dizziness" were also seen, suggesting a low abuse liability in these non-drug using subjects.

Delivered doses from the inhaler are small and produce correspondingly low plasma concentrations. Using an in-vitro system, data available in the PDR and the Federal Register suggests that [3,10], adults who inhale twice in each nostril every two hours may expect a total inhaled dose of 1.9 – 7.2 mg l-methamphetamine in a 24-hour period, or 40 μg to 150 μg per 800 mls of air. However, published source data are not cited and none could be found after an extensive literature search. Using similar dosing assumptions to the PDR we estimate that about 0.2 mg per 24 hours [4.2 (3.3) μg/inhalation] is delivered. Therefore, our estimate is 10-fold less, probably due to differences in the technique used to deliver inhaled dose. Our estimate is based on the renal excretion of l-methamphetamine following controlled dosing in humans, which may have caused considerable variability in our estimates. Heavy use of the inhaler can produce substantial urinary methamphetamine concentrations, some greater than 2000 ng/ml with concentrations up to 6000 ng/ml reported [11]. Poklis (1995) treated 6 subjects either hourly for 3 days or every 2 hours for 5 days while awake. Use of the inhaler every 2 hours did not produce positive urine tests but when administered hourly two subjects had urinary concentrations of 1530 and 1560 ng/ml [12].

The small changes seen on most cardiovascular measures appear to be of little clinical significance. Peak increases in systolic blood pressure of approximately 12 mmHg did occur in some cases in the 16 and 32 inhaler condition. In the 64-inhalation condition systolic blood pressure fell, suggesting a biphasic cardiovascular response to l-methamphetamine. Morgan's report (1979) that the cardiovascular system is more affected by l-amphetamine than d-amphetamine may lead one to expect a similar or greater cardiovascular response from the l-methamphetamine contained in the Vicks^® ^inhaler [13]. However, a 20% fall in mean arterial pressure was seen after 1 mg/kg l-methamphetamine in Sprague-Dawley rats [14]. This was accompanied by a 35% increase in cerebral vascular resistance and a 40% decrease in cerebral blood flow. d-Methamphetamine is usually a potent sympathomimetic alpha agonist and vasoconstrictor where systolic and diastolic blood pressure increase significantly with slight decreases in heart rate consistent with a baroreceptor response [6,15-17]. In contrast, our studies showed that these effects did not occur in response to inhaled or intravenous l-methamphetamine [16].

The small declines in stroke volume and cardiac output at 15 minutes within each period suggest that l-methamphetamine may have some cardiodepressant effects, similar to those seen by Abassi [14]. Heart rate did not change significantly but SVR increased by ~10%, maintaining blood pressure. All subjects in this study were young and had normal cardiovascular function. For people with compromised ventricular function the negative inotropic effects of inhaled l-methamphetamine could become clinically significant; to date no reports of heart failure associated with l-methamphetamine have been reported. In contrast to the nasal decongestants removed from the market the product containing l-methamphetamine modestly increased blood pressure. However, the ~12 mmHg maximal increases seen are unlikely to produce medical complications in young, otherwise healthy people.

The response to exercise was not affected by l-methamphetamine. Even at 4 times the recommended dose, intranasal l-methamphetamine did not alter the effects of exercise. For instance, as measured by impedance cardiography changes in cardiac output (0.5 l/min) and stroke volume (5 mls) in the 32 and 64 inhalation conditions were small. Athletes that may use this product would unlikely have enhanced cardiovascular performance, even at high doses.

Recently, we reported on isomeric differences between d- and l methamphetamine in humans [16]. d-Methamphetamine produces larger and more sustained cardiovascular and subjective effects than identical doses (0.5 mg/kg and 0.25 kg/kg i.v. over 1 minute) of l-methamphetamine, suggesting that the enantiomers act through different pharmacologic mechanisms. In this study, intranasal l-methamphetamine generally did not produce clinically significant cardiovascular effects. Small increases in visual analog ratings of "Any Drug Effect" indicate that subjects perceived effects and small increases in "Bad Drug Effect" and "Dizziness" suggest non-drug users do not find inhaling l-methamphetamine pleasurable. The 64-inhalation condition produced a small (change score of ~6) increase in "Good Drug Effect" suggesting a low potential for abuse even though occurrences of inhaler abuse is reported in the literature [1,18,19]. Larger doses of intravenous l-methamphetamine are psychoactive and may have some abuse potential in methamphetamine users [16].

Stroke is a serious but rare complication of nasal decongestant use. The risk of stroke after phenylpropylalinine (a related nasal decongestant now off the market) was greatest in those just initiating use and may be related to the development of tolerance with repeated use [4]. Methamphetamine is an indirectly acting sympathomimetic amine that potentiates the presynaptic release and blocks reuptake of catecholamine neurotransmitters (norepinephrine and dopamine), thus activating the sympathetic nervous system [20-22]. However, sustained exposure to high doses of methamphetamine results in depletion of monoamine [22-25]. Infrequent users of the intranasal l-methamphetamine (people with a cold) may have more catecholamine stores compared to chronic methamphetamine abusers, and could have a greater pharmacodynamic response to small doses of l-methamphetamine. According to Zhu et al. (2000) stroke is the most common cause of sudden death in first time methamphetamine users [26]. Our results suggest this adverse event should be uncommon with inhaled l-methamphetamine.

There are several limitations to our study. The lack of a placebo limits our ability to assess the importance of the small increases in blood pressure seen. Similar diurnal changes in blood pressure may have occurred without l-methamphetamine. We only studied normotensive people; results in hypertensives might differ. Normotensives may be more resistant to alpha agonist stimuli; in this study they had minimal responses to both intravenous l-methamphetamine and phenylephrine. Hypertensives might show more cardiovascular effects after a sympathomimetic amine like l-methamphetamine, and possibly be at greater risk for cardiovascular adverse events.

In summary, the low doses of inhaled l-methamphetamine delivered from the Vicks inhaler produced little cardiovascular effects in healthy people, even when given in amounts much larger than recommended. Over time, changes in blood pressure and heart rate were clinically insignificant. Significant peak increases in systolic blood pressure did occur, however, a biphasic response was seen since systolic blood pressure fell following the highest inhaler dose. The clinically significance of this finding requires further study. Stroke volume and cardiac output decreased but systemic vascular resistance increased suggesting a compensatory mechanism to maintain blood pressure. Overall, the results indicate that inhaled l-methamphetamine at these doses appears to be a slight cardiodepressant. A straightforward dose-dependence did not occur on most measures. The mild subjective changes suggest that l-methamphetamine, at least when delivered from a widely available non-prescription product, is well tolerated and has a low potential for abuse.

## Pre-publication history

The pre-publication history for this paper can be accessed here:


